# Persistent Left Superior Vena Cava: Unanticipated Stumbling Block in Cardiac Resynchronization Therapy

**DOI:** 10.7759/cureus.68136

**Published:** 2024-08-29

**Authors:** Shodhan Aithal, Anunay Gupta, Sandeep Bansal, Hermohander S Isser, Puneet Gupta

**Affiliations:** 1 Cardiology, Vardhman Mahavir Medical College and Safdarjung Hospital, Delhi, IND

**Keywords:** heart failure, heart failure with reduced ejection fraction, left superior vena cava (lsvc), cardiac resynchronization therapy (crt), permanent pacemaker implantation (ppm)

## Abstract

Persistent left superior vena cava (PLSVC) is a relatively rare anatomical anomaly, with a higher prevalence in those with congenital heart defects. While typically asymptomatic, its presence can complicate certain medical procedures, particularly cardiac interventions, such as the implantation of cardiac resynchronization therapy (CRT) devices, due to acute angulation. In this report, we discuss the challenges posed by the unanticipated presence of PLSVC during CRT device implantation and describe the technique used for lead placement using Judkins Right catheter for support, placing coronary wire, and later placing the left ventricle (LV) lead with the help of buddy wire technique, resulting in successful insertion of all three CRT leads despite the anatomical challenges.

## Introduction

Heart failure with reduced ejection fraction (HFrEF) is a prevalent condition associated with significant morbidity and mortality. Dilated cardiomyopathy (DCM) is a common etiology of HFrEF characterized by ventricular dilation and systolic dysfunction. Left bundle branch block (LBBB) is frequently observed in patients with HFrEF and is associated with further deterioration of cardiac function and poor prognosis. In such cases, optimizing therapeutic interventions is paramount to improving patient outcomes. Cardiac resynchronization therapy (CRT) with pacemakers (CRT-P) and/or implantable cardiac defibrillators (ICD-CRT-D) has emerged as a valuable treatment modality for selected patients with HFrEF and LBBB [1].

Persistent left superior vena cava (PLSVC) is a congenital anomaly characterized by the presence of a left-sided superior vena cava (SVC) that persists in addition to or instead of the right-sided SVC. A communicating vein is also reported [2]. The prevalence is 0.3-2% in the general population and is deemed to be higher in the presence of congenital anomalies [2,3]. While often asymptomatic and found accidentally [4], PLSVC can complicate certain medical procedures, particularly those involving central venous access or cardiac interventions. The implantation of CRT devices, which involves the placement of leads in the right ventricle (RV) and coronary sinus (CS), may present technical challenges in the presence of PLSVC [5]. Here, we discuss the management of PLSVC encountered during CRT device implantation.

## Case presentation

**Figure 1:**
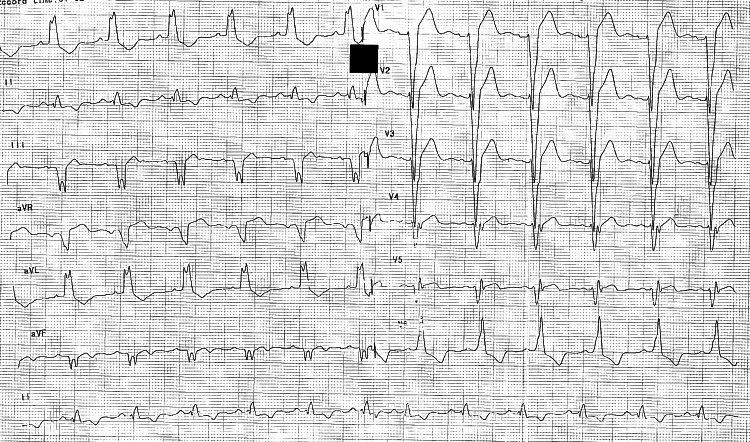
Baseline ECG with typical LBBB Baseline ECG showing typical LBBB with notching in avL, with left axis deviation and a wide QRS duration of 180 ms. LBBB, left bundle branch block

A 46-year-old female with no prior comorbidities presented with worsening dyspnea on exertion for one year. Electrocardiography showed a typical LBBB with a wide QRS of 180 ms, as depicted in Figure 1.

Echocardiography revealed severe left ventricular systolic dysfunction with an ejection fraction of 20% with global left ventricular hypokinesia, moderate mitral regurgitation, and evidence of ventricular dyssynchrony. Coronary angiography revealed normal epicardial coronary arteries. The patient was diagnosed to have DCM and was optimized on guideline-directed medical therapy but remained symptomatic despite providing a maximum tolerable dose.

Given the persistence of symptoms despite optimal medical therapy, the decision was made to proceed with CRT implantation. The patient was taken to the catheterization lab for the procedure, and under fluoroscopic guidance, a left-arm venogram and CS venogram were performed. This revealed the presence of PLSVC draining into the CS, as demonstrated in Videos 1, 2.

**Video 1:** Venogram depicting PLSVC with no communicating branch PLSVC, persistent left superior vena cava

**Video 2:** Extension of venogram demonstrating continuation of PLSVC to the CS and draining to RA PLSVC, persistent left superior vena cava; RA, right atrium; CS, coronary sinus

A left deltopectoral incision followed by dissection was carried down to the pre-pectoral fascia. Axillary vein access was obtained using a modified Seldinger technique. Subsequently, a multipurpose sheath was advanced into the CS over the guide wire via LSVC. A selective venogram confirmed the upward course of the posterolateral vein (PL). Judkins Right (JR) 5F guiding catheter was advanced through the sheath to the level of angulation; PL was hooked, and a Whisper Extra Support guide wire was then navigated deep into the PL, as depicted in Video 3.

**Video 3:** Selective hooking of PL with the help of JR 5 catheter JR, Judkins Right; PL, posterolateral vein

Despite encountering difficulty with lead advancement due to the acute bend, successful positioning of the quadripolar left ventricular lead was achieved using another coronary wire for the buddy wire technique for extra support. The final left ventricular lead was confirmed after achieving adequate lead parameters and stability. Phrenic nerve capture was carefully assessed and avoided at high outputs.

**Figure 2:**
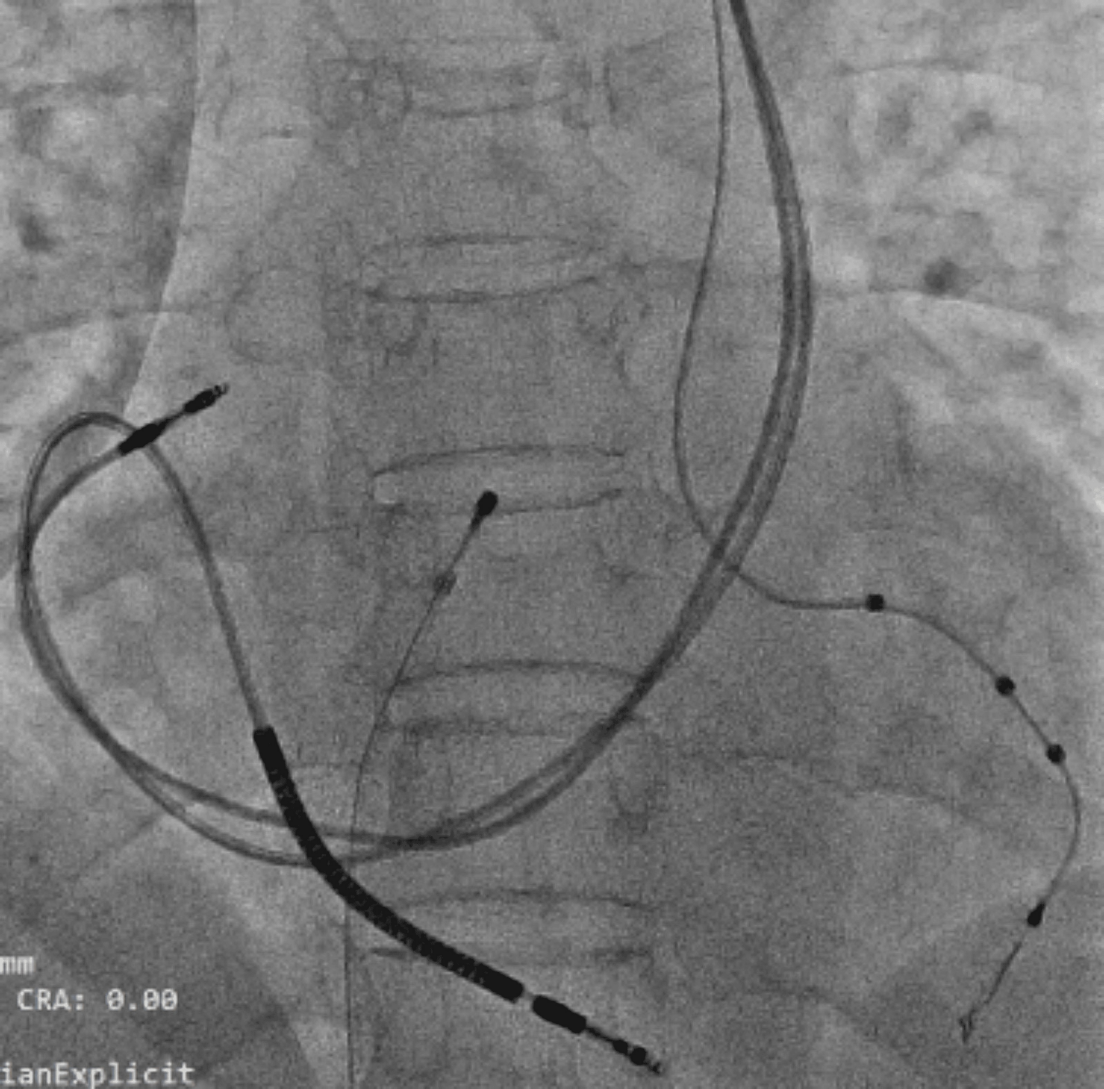
Final position of LV, RV, and RA leads in the AP view Quadripolar LV lead is placed in the posterolateral vein, defibrillator lead is placed in the RV after taking a sharp acute bend after its entry from CS, and RA lead is placed in the right atrial appendage. LV, left ventricle; RV, right ventricle; RA, right atrium; AP, anteroposterior; CS, coronary sinus

The right atrial (RA) and RV leads were placed subsequently. There was difficulty in positioning the RV lead due to acute angulation from CS to RV, which could be negotiated after providing an appropriate curve to the stylet, as demonstrated in Figure 2. The parameters of all three leads were acceptable, the CRT device was secured, and the wound was closed with sutures.

**Figure 3:**
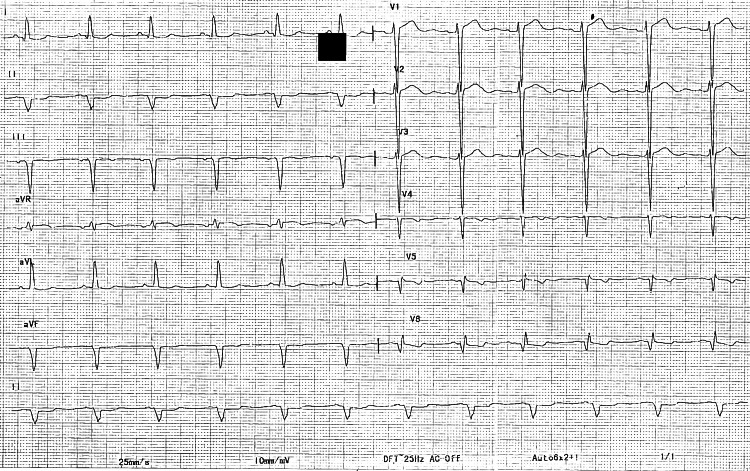
Post-CRT ECG (QRS, 110 ms) The optimization of CRT with LV pacing was followed by RV pacing with a delay of 45 ms, resulting in a narrow QRS of 110 ms with a positive initial vector in lead v1. CRT, cardiac resynchronization therapy; LV, left ventricle; RV, right ventricle

Post-implantation fluoroscopy and electrocardiography confirmed appropriate lead placement. Post-implantation ECG is depicted in Figure 3.

The patient tolerated the procedure well without any immediate complications. She was discharged home on post-procedural day five.

## Discussion

PLSVC is a rare anatomical variant with an estimated incidence of 0.3-2% in the general population [2]. While often asymptomatic, its presence may complicate central venous access and cardiac interventions. Technical difficulties encountered while implanting leads in PLSVC are abnormal venous anatomy, abnormal access to the heart, associated congenital anomalies, absence of bridging innominate vein between right SVC and PLSVC, relatively rapid blood flow in the dilated CS, negotiation of RV lead across tricuspid valve with acute bend, and inability to reach RA appendage, necessitating placement in RA free wall [6-9]. In the context of CRT implantation, PLSVC requires careful consideration to ensure appropriate lead placement and optimal device function.

In cases where PLSVC is encountered, several strategies can facilitate lead placement. Utilizing pre-procedural imaging, such as venography, can help identify the anatomy and plan the approach accordingly [10]. In some cases, switching to the contralateral side for lead placement may be necessary [10,11]. Accessing the CS via the PLSVC may be feasible, allowing for lead placement without the need for switching sides. Modifications to lead delivery systems and catheter manipulation techniques may aid in navigating the venous anatomy and achieving optimal lead positioning. Curved sub-selective catheters and extra support guide wires were also used in a similar case to facilitate lead placement [9]. Few studies have suggested utilizing active lead fixation or a preferential right pectoral approach [11]. With careful planning and improvisation of techniques, CRT device implantation can often be completed successfully from the left side.

In our case, successful CRT implantation was achieved despite the presence of PLSVC. Pre-procedural imaging, including venography, played a crucial role in identifying the venous anatomy and guiding lead placement. Fluoroscopy and electrocardiography allowed for real-time assessment of lead position and electrical parameters. The utilization of supporting guide catheters and additional guide wires helped stabilize the left ventricle (LV) lead and navigate it across the acute bend at the origin of the posterior LV. Shaping the stylet to facilitate the RV lead positioning is essential.

## Conclusions

This case demonstrates successful CRT implantation via the left pectoral approach in a patient with anatomical complexity of PLSVC, highlighting the feasibility and importance of the modified approach to navigate anatomical challenges. Familiarity with alternative techniques like the usage of additional support guiding catheters, initial placement of coronary wire, and utilization of buddy wire techniques are essential for achieving optimal outcomes in patients with complex venous anatomy undergoing CRT implantation. This case report highlights that it is not necessary to use opposite-site access for the successful implantation of CRT leads, which can be achieved on the same access site with reasonable alterations in the implantation technique. Further research and technological advancements may help refine strategies for lead placement in such cases.
